# Effect of Community Engagement Interventions on Patient Safety and Risk Reduction Efforts in Primary Health Facilities: Evidence from Ghana

**DOI:** 10.1371/journal.pone.0142389

**Published:** 2015-11-30

**Authors:** Robert Kaba Alhassan, Edward Nketiah-Amponsah, Nicole Spieker, Daniel Kojo Arhinful, Alice Ogink, Paul van Ostenberg, Tobias F. Rinke de Wit

**Affiliations:** 1 Amsterdam Institute for Global Health and Development, University of Amsterdam, Amsterdam, Netherlands; 2 Department of Epidemiology, Noguchi Memorial Institute for Medical Research, University of Ghana, Legon, Accra, Ghana; 3 Department of Economics, University of Ghana, Legon, Accra, Ghana; 4 PharmAccess Foundation, Amsterdam, Netherlands; 5 Joint Commission International (JCI), Chicago, Illinois, United States of America; Kenya Medical Research Institute—Wellcome Trust Research Programme, KENYA

## Abstract

**Background:**

Patient safety and quality care remain major challenges to Ghana’s healthcare system. Like many health systems in Africa, this is largely because demand for healthcare is outstripping available human and material resource capacity of healthcare facilities and new investment is insufficient. In the light of these demand and supply constraints, systematic community engagement (SCE) in healthcare quality assessment can be a feasible and cost effective option to augment existing quality improvement interventions. SCE entails structured use of existing community groups to assess healthcare quality in health facilities. Identified quality gaps are discussed with healthcare providers, improvements identified and rewards provided if the quality gaps are closed.

**Purpose:**

This paper evaluates whether or not SCE, through the assessment of health service quality, improves patient safety and risk reduction efforts by staff in healthcare facilities.

**Methods:**

A randomized control trail was conducted in 64 primary healthcare facilities in the Greater Accra and Western regions of Ghana. Patient risk assessments were conducted in 32 randomly assigned intervention and control facilities. Multivariate multiple regression test was used to determine effect of the SCE interventions on staff efforts towards reducing patient risk. Spearman correlation test was used to ascertain associations between types of community groups engaged and risk assessment scores of healthcare facilities.

**Findings:**

Clinic staff efforts towards increasing patient safety and reducing risk improved significantly in intervention facilities especially in the areas of leadership/accountability (Coef. = 10.4, p<0.05) and staff competencies (Coef. = 7.1, p<0.05). Improvement in service utilization and health resources could not be attributed to the interventions because these were outside the control of the study and might have been influenced by institutional or national level developments between the baseline and follow-up period. Community groups that were gender balanced, religious/faith-based, and had structured leadership appeared to be better options for effective SCE in healthcare quality assessment.

**Conclusion:**

Community engagement in healthcare quality assessment is a feasible client-centered quality improvement option that should be discussed for possible scale-up in Ghana and other resource poor countries in Africa.

## Introduction

Patient safety and quality healthcare are critical dimensions of universal health coverage (UHC) yet remain complex challenges to health systems globally [1]. Since the launch of the World Health Organization (WHO) Patient Safety Programme (PSP) in 2004, over 140 countries globally (including Ghana) agreed to intensify efforts towards addressing challenges relating to unsafe and poor quality healthcare through established science-based systems.

Although patient safety and patient-centered care are recognized as important requirements for meeting patient needs [2,3], achievements in this regard remain modest especially in resource constrained countries in sub-Saharan Africa (SSA). In the light of these resource constraints, it is imperative that available community resources are harnessed through systematic community engagement (SCE) to complement central government’s efforts towards quality care improvement [4,5,6]. Community engagement in health has proved effective in empowering communities/clients, harnessing local resources, enhancing service provider accountability and experiences of clients with healthcare providers [7–11].

Morgan and Lifshay [12] defined community engagement in the context of public health as dynamic relationships and dialogue between community members and local health professionals with varying degrees of community and higher level health authorities’ involvement in decision-making and control. In the context of this study, SCE is defined as structured involvement of existing community groups and associations in monitoring service quality and engaging with relevant stakeholders on a common platform towards addressing identified quality care gaps.

Even though community engagement in health is not an entirely new concept in Ghana [7,11,13–15] it is yet to be adopted as a quality improvement strategy. Limitations of conventional quality assessment methods such as patient satisfaction surveys, institutional peer reviews, exit interviews and mystery client shopping [16–20] demand that complementary strategies such SCE in quality healthcare assessment are explored. Unlike the conventional methods, SCE is more community-focused, less technical, and potentially less expensive with higher sustainability prospects because it is mainly championed by community members and resources largely mobilized from within the community.

The SCE interventions, evaluated in this paper, were designed to systematically engage existing community groups and associations in assessing healthcare quality in health facilities accredited by the National Health Insurance Authority (NHIA) in Ghana. The NHIA is the regulatory body under the Ministry of Health (MoH) responsible for accreditation of health facilities willing to render services to subscribers of Ghana’s National Health Insurance Scheme (NHIS).

This paper evaluates impact of the SCE interventions on patient safety and risk reduction efforts (i.e. proxies for healthcare quality indicators) in the intervention health facilities. The main research hypothesis is that patient safety and risk reduction efforts will improve significantly in facilities that received the SCE interventions than those that did not, controlling for facility human/material resource capacity, location and ownership. Policy implications of the evaluation outcome are also discussed.

## Methodology

### Study design and setting

A Randomized Controlled Trial (RCT) was conducted in 64 NHIS-accredited clinics/health centres in the Greater (n = 32) and Western (n = 32) regions of Ghana. RCT was deemed appropriate for this study because it is one of the most scientifically rigorous methods of hypothesis testing [21] and the gold standard trial for evaluating effectiveness of interventions while preventing selection bias [22,23].

### Randomization and Sampling procedure

The sample frame was primary healthcare facilities (i.e. clinics and health centres) accredited by the NHIA. Clinics/health centres were sampled because they are relatively less complex and could easily be monitored for impact of the implemented interventions. A total of 16 NHIS district offices (used as proxy administrative districts) were sampled at random (i.e. 8 from each region); next, four (4) health facilities around the catchment area of each NHIS district office were also randomly sampled.

In each of the study regions, 16 health facilities were randomly assigned to receive treatment (i.e. SCE) and another 16 assigned as controls (i.e. no SCE). Random allocation of the health facilities into the intervention and control arms of the project was conducted such that in each district, the names of all 4 health facilities were written on pieces of paper. Subsequently, for each district at a time, two ballots (representing health facilities) were randomly picked without replacement to receive intervention. Per this criteria 32 health facilities and their catchment area were randomly assigned as intervention facilities and the remaining 32 as controls (see S1 Fig).

### Statistical power and representativeness of the study

The sample size of 64 clinics is deemed representative because the number was determined by 80% statistical power calculation needed to attain up to 1,920 households and over 9,000 individuals around the catchment area of these 64 clinics. This statistical power is required to determine true effect of implemented interventions and increase the probability that the study correctly rejects the null hypothesis (H_0_) that the SCE interventions had no significant effect on any of the healthcare quality proxies (i.e. 5 risk areas) [24]. Moreover, the 64 facilities constitute about 5% of the approximately 1,180 accredited clinics/health centres in Ghana while the 32 intervention facilities represent about 10% of the total number of accredited clinics/health centres in each of the two study regions [25].

### Implementation of the Systematic Community Engagement (SCE) Interventions

The SCE interventions were implemented for nearly one year (from June, 2013 to March, 2014). Two categories of SCE were implemented namely: *MyCare* (also called Intensive Engagement) and Light Engagement (LE). The LE intervention used existing community groups/associations to identify service delivery gaps in healthcare facilities and NHIS district offices. The *MyCare* component engaged clients and relevant stakeholders in a participatory process; the focus was on individual clients contrary to the group approach in the LE. Both categories of interventions were implemented and evaluated concurrently.

This paper emphasizes the LE arm of the SCE because the MyCare intervention was implemented in only 6 out of the 32 intervention facilities; moreover, facilities where *MyCare* was implemented, the LE component was also implemented. In this paper, the SCE thus refers specifically to the LE interventions.

### Light Engagement implementation steps

**Step 1:** The first step involved recruitment and training of 52 facilitators, and identification of existing community groups/associations. One facilitator was assigned to each of the of 52 community groups in the two study regions (26 in each region).

Eligibility criteria for selection of community groups included: (i) documented evidence of routine meetings (at least four times a year), (ii) regular meeting venue, (iii) clear leadership structure, (iv) non-partisan and (v) active membership not less than an intuitive number of ten (10). These criteria were meant to ensure the groups are active in their activities and reasonably represent cross-section of community opinion. Where more than two community groups around the catchment area of a health facility met these eligibility criteria, simple random sampling was done to select two groups to control bias.

Even though the types of community groups varied based on core activities, these features did not systematically relate to particular features of the sampled clinics. The five (5) eligibility criteria were used as the basis for selecting the community groups to avoid engaging groups that systematically share features with the clinics they assess. A pairwise correlation test confirmed that many community groups’ features did not significantly associate with clinic characteristics (see S1 Table).

The community groups recruited comprised of 22 religious/faith-based groups; 8 traders groups; 1 widows group; 3 community volunteers groups; 3 musician groups; 5 artisans groups and 11 youth groups. Average group size during assessment was 29 members (SD = 20). More than half of the groups were female dominated, 13 were male dominated; 2 were all males; 5 were all females, and 1 was balanced males and females. Approximately 56% of the groups were a combination of literates and illiterates; 23% were mainly literates, and 21% mainly illiterates. In terms of age, 65% of the groups had predominantly elderly members (31^+^ years) and 35% had predominantly youthful members (18–30 years).

**Step 2:** The second engagement step entailed a first round of community group assessment of healthcare quality based on group members’ most recent (at most 6 months) experiences with the particular intervention health facility in their community. Healthcare quality proxies used to guide community members during assessment were: (1)staff attitude, (2)punctuality to work, (3)client waiting time, (4)queuing system, (5)availability of drugs, (6)information provision to clients, (7)equal treatment for insured and uninsured clients, (8)complaint system for clients, (9)client-provider communication, and (10) net promoter score (NPS). The NPS is an indicator used to determine the possibility of the healthcare client recommending the health facility to a fellow client (e.g. relative, friend or co-worker) based on their personal experiences of the quality of health service delivery. The rating is done on a five-point Likert scale from 1 = “Very disappointing” to 5 = “Very satisfactory”.

During engagement sessions, group members rated performance of their nearest health facility on these quality care proxies on a five-point Likert scale from 1 = “Very disappointing” to 5 = “Very Satisfactory”. The group assessments were conducted in the communities to avoid possible bias and client intimidation. Anonymity of group members was assured by reporting group perception ratings without members’ personal details. The average meeting duration per group was 41 minutes (SD = 13.8).

**Step 3:** The third implementation step entailed regional level validation and feedback sessions to disseminate the group assessment findings with facility heads, clients and NHIA representatives. This platform provided the service providers the opportunity to recognize and accept gaps in healthcare quality and agree on quality improvement plans with timelines and responsible persons.

**Step 4:** During the fourth step, facilitators followed-up on the service providers (3 months after validation and feedback sessions) to ascertain whether or not providers were implementing the agreed action plans towards quality improvement.

**Step 5:** The last step rewarded best performing health facilities after a second round of community assessment (approximately six months after the first assessment). A citation plaque of honor and a token financial incentive of GHC 1,000.00 (Ghanaian Cedis), approximately US$ 280.0 was awarded to one best performing facility in a district to encourage competition among peers towards quality improvement (see S2 Fig).

### Statistical analysis

Names of healthcare facilities sampled for the study were coded after data cleaning to ensure anonymity during data analysis. Parameters used to ascertain patient safety and risk status (i.e. healthcare quality proxies) were five primary risk areas defined in an assessment tool kit called *SafeCare Essentials*; 41 assessment criteria make up the five risk areas and each criteria is scored on four levels of effort (0–3) where high levels depict better efforts by staff of that pertinent health facility towards enhancing patient safety and reducing risk.

The five primary risk areas which are also the main outcome variables of interest are: leadership and accountability (7 criteria); competent and capable workforce (7 criteria); safe environment for staff and patients (10 criteria); clinical care of patients (10 criteria), and improvement of quality and safety (7 criteria) [26,27].

Before and after the SCE interventions, two trained research assistants did double scoring per healthcare facility and later reconciled scores using a Personal Device Assistant (PDA). Field data was then uploaded to a web-based platform called *AfriDB* for validation and approval by a PharmAccess quality assessment team in Amsterdam, Netherlands. The *SafeCare Essentials* tool is provided by the SafeCare Initiative, a collaboration of the PharmAccess Foundation, the Council for Health Services Accreditation of Southern Africa (COHSASA) and the Joint Commission International (JCI) [27].

The tool is designed to rapidly assess patient safety and risk status according to staff level of efforts. These levels of effort are not quality indicators *per se* but meant to ascertain whether patient safety activities in pertinent health facility was random and unsustainable when present or if there are sufficient structures and processes in place to attain its quality care aspirations. The tool identifies the activities, behaviors, policies or processes that must be in place to score at the next higher level.

For the purposes of our analysis, mean percentage (%) scores were computed for each of the sampled health facilities based on their scores on the 41 criteria. For every health facility the mean % scores were computed by summing all applicable criteria scores (0–3) under each risk area divided by the total expected score per risk area and multiplied by 100. Thus if a health facility scores 3 in all 10 criteria under leadership and accountability, the mean% score for that risk areas will be 30 (actual score) divided by 30 (total expected score) and multiplied by 100 (i.e. 100%). This computation is to ensure the outcome variables of interest (the five risk areas) are reported as continuous variables and are easy to interpret.

The SafeCare Essentials tool is deemed appropriate for the Ghanaian and African context because it has been validated in over 2,000 health facilities in Ghana, Nigeria, South Africa, Kenya, Mozambique and Namibia [28] prior to its application in this study.

All analyses were done on “intention to treat” basis [22] and comparison of pre and post intervention quality scores was done using the paired *t-test*. Since the intervention and control health facilities were comparable in several respects per the RCT design, propensity score matching to estimate treatment effect was not relevant [22]. Instead, multivariate multiple regression analysis was performed to determine the effect of the SCE interventions on the five primary risk areas.

Covariates controlled for were: health facility material/human resource capacity, location (rural/urban) and ownership (private/public) following a multicollinearity test. Post estimation predictive margins and contrast tests were done to determine the interactive effect of variables on the five primary risk areas. Reported p-values are two-tailed test of hypothesis and p<0.1, p<0.05 or p<0.0001 are considered statistically significant.

Spearman rank correlation test was used to ascertain the associations between the different types of community groups and assessment scores of health facilities on the five primary risk areas after the interventions. Statistical analyses were performed using Stata version 12.0 (StataCorp, College Station).

### Ethical clearance

Ethical clearance for this study was sought from the Ghana Health Service (GHS) Ethical Review Committee (ERC) (Clearance number: GHS-ERC: 18/5/11). Moreover, written informed consent was sought from individual key informants, management of the healthcare facilities, district health directorates and district NHIA offices.

## Results

### Background information of health facilities

Only facilities with complete follow-up and baseline data were included in the analysis for paired comparison purposes. One health facility in the control group in Greater Accra region was lost to follow-up in 2014 reducing the follow-up sample size to 63 (59% private and 41% public). The number of private health facilities that received intervention was 21 and 16 were controls; 11 public facilities received intervention and 15 were controls. In terms of facility geographic location, 18 rural facilities received intervention and 18 were controls; 14 urban facilities received intervention and 13 were controls.

**Fig 1:**
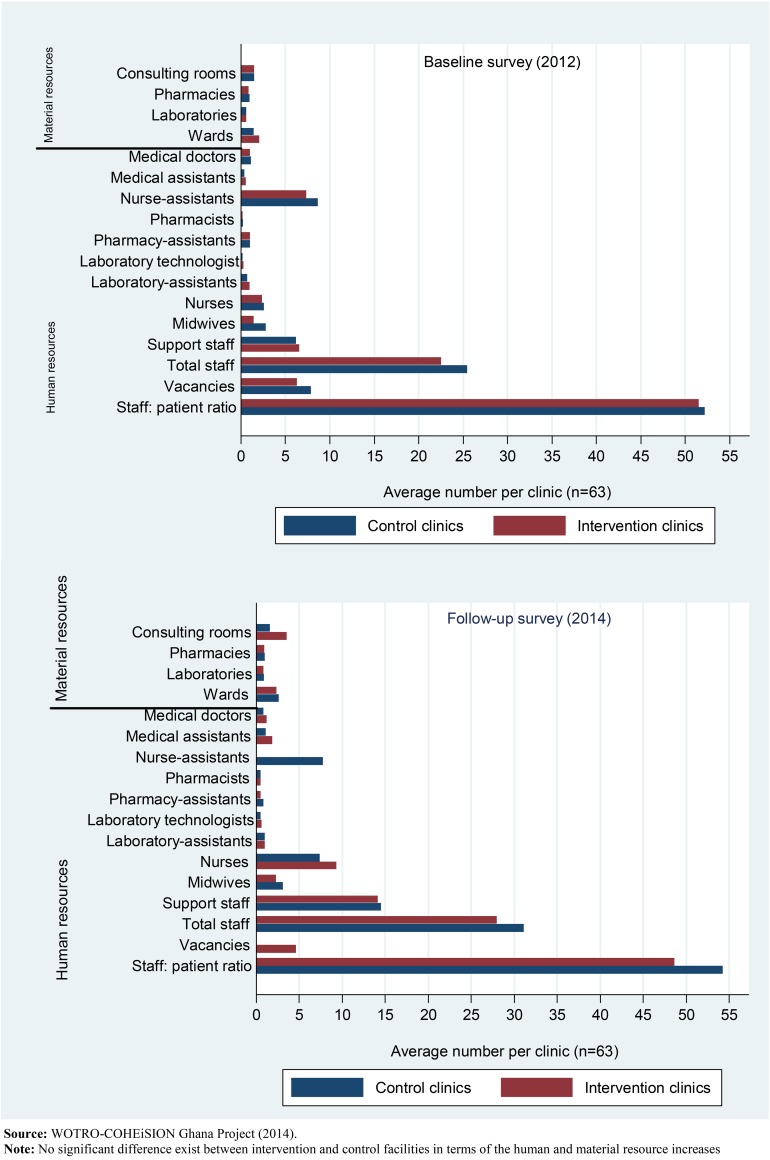
Human and material resources in intervention and control facilities in 2012 and 2014.

As shown in Table 1 the average human and material resource situation per health facility appeared to have improved during the follow-up survey. Significant increases were recorded in the number of nurses, laboratory technologists, pharmacists and support staff (p<0.05); likewise, though the average number of wards and laboratories per clinic increased significantly at follow-up (p<0.05) these increases may not be associated with the interventions because they were not within the control of the study (see Fig 1).

**Table 1:** Background information of health facilities before and after interventions.

Resources per clinic		Baseline (2012)	Follow-up (2014)	Stat. Diff	
*Staff*	^+^No. Clinics	Mean (SD)	Mean (SD)	Mean Diff.	p-value
Medical doctors	10	0.9(1.0)	1.0(1.0)	0.1	0.5911
Medical assistants	50	0.5(0.5)	1.5(2.3)	1.0	0.0020†
Professional nurses	44	2.4(3.7)	8.4(10.7)	6.0^++^	0.0002†
Midwives	58	2.2(3.2)	2.7(3.3)	0.5	0.0584*
Pharmacists	46	0.2(0.6)	0.5(0.8)	0.3	0.0090†
Pharmacist-assistants	42	0.9(1.4)	0.7(1.6)	-0.2	0.2862
Laboratory technologists	44	0.3(0.1)	0.6(0.8)	0.3	0.0035†
Laboratory technicians	54	0.9(1.3)	1.0(1.1)	0.1	0.5602
Support staff	61	6.5(8.8)	14.3(13.7)	7.8^++^	0.0000‡
Total staff	63	24.0(21.8)	29.5(26.1)	5.5^++^	0.0059†
Total staff to patient ratio	63	51.9(33.1)	51.4(37.5)	-0.5	0.9278
Total staff vacancies	6	4.8(4.7)	2.3(3.8)	-2.5^++^	0.4438
***Health infrastructure***					
Admission/observation wards	63	1.8(1.0)	2.5(1.3)	0.7	0.0000‡
OPD consulting rooms	63	1.5(1.2)	2.6(7.1)	1.1	0.2528
Pharmacies	60	1.0(0.2)	1.0(0.3)	0.0	0.5681
Medical laboratories	50	0.7(0.5)	0.9(0.4)	0.2	0.0036†

**Source:** WOTRO-COHEiSION Ghana Project (2014); paired t-test *p<0.1

†p<0.05

‡p<0.0001

**NOTE:** All means and SD are rounded up to one decimal place

^+^Number of clinics with complete comparable baseline and follow-up data

^++^The phenomenal increment in the staffing situation of health facilities during follow-up may not be attributed to the SCE interventions since the project did not have the capacity to influence recruitment of additional staff. Perhaps these increases could best be ascribed to institutional and national level developments between the baseline and the follow-up surveys (approximately 2 years). Moreover, the upgrading of a number of the health facilities from clinic/health centre status to hospital status by the NHIA might have influenced these statistics since such upgrading often require a commensurate improvement in human and material resource capacity.

**Fig 2:**
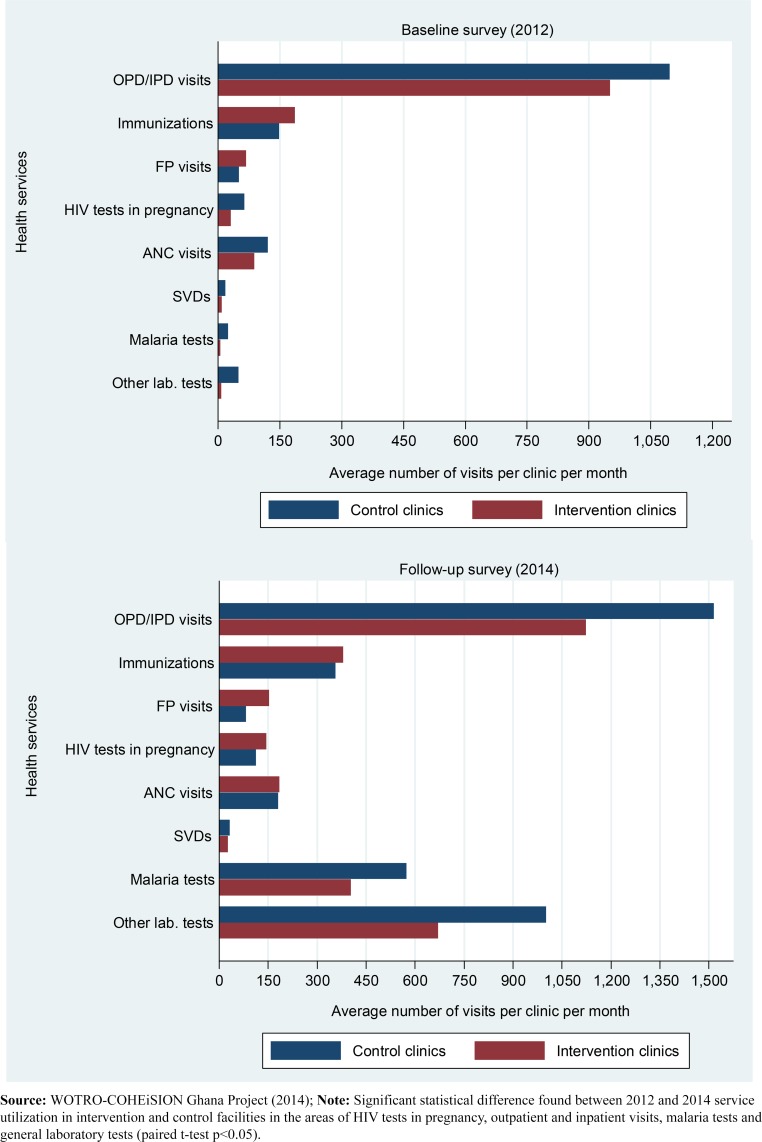
Service utilization in intervention and control facilities in 2012 and 2014. **Legend:** OPD (outpatient department); IPD (Inpatient department); FP (Family planning); SVDs (Spontaneous vaginal deliveries); HIV (Human Immuno-deficiency virus); ANC (antenatal care).

**Fig 3:**
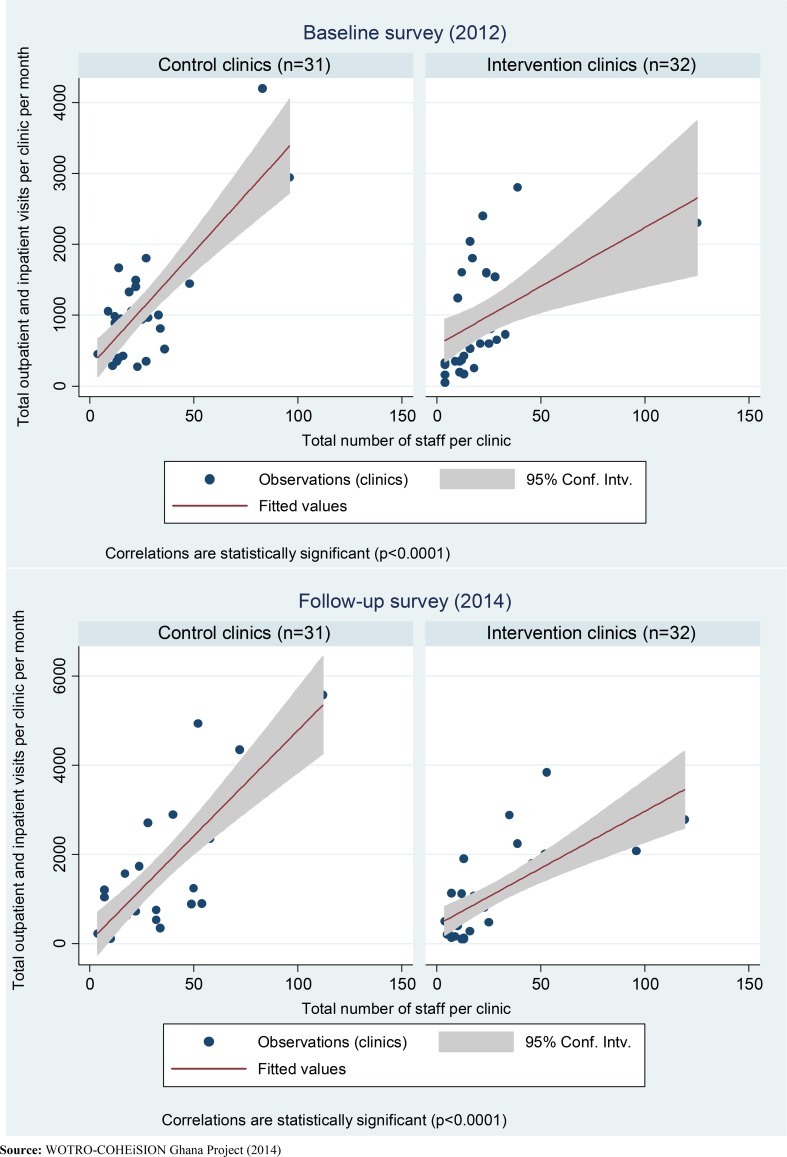
Association between health resources and service utilization in 2012 and 2014.

The results also show that average health service utilization (i.e. number of client visits for health services) per health facility per month increased significantly between 2012 and 2014. Between 2012 and 2014, intervention facilities recorded significant increases in the average number of HIV tests in pregnancy per month (from 50 to 143; p = 0.0167) while control facilities recorded improvement in number of outpatient and inpatient visits per month (from 1,096 to 1,516; p = 0.0319). The average number of laboratory tests per clinic per month increased by over 97% in intervention facilities (from 10 to 390) compared to 93% in control facilities (from 58 to 803) (p<0.05); similarly, the number of malaria tests increased by approximately 98% in intervention facilities (from 6 to 285) compared to 92% in control facilities (from 29 to 375) (p<0.05) (see Figs 2 and 3).

### Effect of SCE interventions on patient safety and risk reduction efforts in clinics

**Fig 4:**
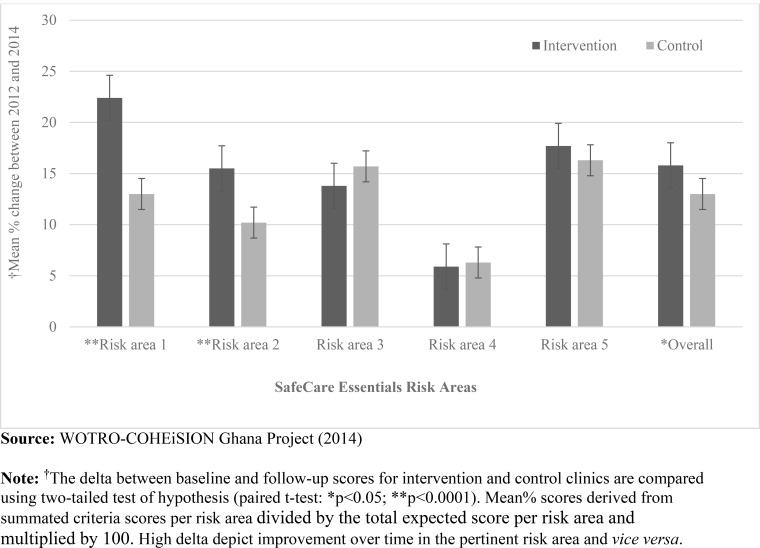
Error bars showing average deltas in patient risk areas between 2012 and 2014. **Legend:** Risk area 1 (Leadership processes and accountability); Risk area 2 (Competent and capable workforce); Risk area 3 (Safe environment for staff and patients); Risk area 4 (Clinical of patients); Risk area 5 (Quality improvement and safety).

Assessment scores in risk area 1 (leadership and accountability) show that intervention health facilities recorded relatively higher marginal increases between 2012 and 2014 (mean = 22) than control facilities (mean = 13) (p<0.0001); likewise, intervention facilities (mean = 16) improved more than control facilities (mean = 10) in risk area 2 (i.e. competent/capable workforce) (p<0.0001). Overall score in all risk areas was relatively higher in intervention (mean = 16) than control facilities (mean = 13) (p<0.05). There were no significant differences between intervention and control health facilities in risk areas 3, 4 and 5 which are predominantly medical technical areas (see Fig 4).

Results of a multivariate multiple regression analysis showed that the SCE interventions significantly enhanced leadership processes and accountability. It was found that intervention facilities had 10.4 times higher odds of an improvement in leadership processes and accountability than control facilities (Coef. = 10.4; p<0.05). Intervention facilities also had 7.1 times higher odds of an improvement in staff competencies (Coef. = 7.1; p<0.05) than control facilities. Besides the SCE interventions, number of laboratories per clinic appeared to be a significant determinant of scores in the five risk areas (p<0.05) (see Table 2).

**Table 2:** Association between patient safety markers and SCE interventions: covariates corrected.

			Multivariate			
	Risk area 1	Risk area 2	Risk area 3	Risk area 4	Risk area 5	^+^Overall score
Variables	Coef.(95% CI)	Coef.(95% CI)	Coef.(95% CI)	Coef.(95% CI)	Coef.(95% CI)	Coef.(95% CI)
Intervention clinics	10.4(0.3 20.5)**	7.1(0.31 0.8)**	4.1(-2.7 11.0)	4.4(-1.8 10.5)	5.7(-4.2 15.6)	6.1(-0.96 13.10)*
Control clinics	1.0	1.0	1.0	1.0	1.0	1.0
Private clinics	1.27(-9.9 12.4)	0.10(-7.4 7.6)	4.3(-3.2 11.8)	3.1(-3.8 1.0)	8.4(-2.5 19.3)	3.6(-4.2 11.3)
Public clinics	1.0	1.0	1.0	1.0	1.0	1.0
GAR clinics	-3.4(-14.1 7.40)	-5.0(-12.2 2.2)	0.12(-7.2 7.4)	-4.1(-10.7 2.4)	-4.0(-14.6 6.5)	-3.0(-10.5 4.5)
WR clinics	1.0	1.0	1.0	1.0	1.0	1.0
No. of consulting rooms	-0.10(-0.8 0.56)	-0.03(-0.48 0.4)	-0.38(-0.83 0.08)*	-0.31(-0.7 0.10)	-0.25(-0.910.40)	-0.24(-0.70 0.23)
No. of Laboratories	17.9(4.0 31.5)**	11.4(2.3 0.5)**	8.2(-1.0 17.4)*	8.5(0.11 16.8)**	13.4(.08 26.7)*	11.4(1.86 20.9)**
Staff: patient ratio	0.07(-0.1 0.20)	0.02(-0.07 0.1)	-0.01(-0.10 0.08)	0.02(-0.06 0.10)	0.02(-0.110.15)	0.021(-0.07 0.11)

**Source:** WOTRO-COHEiSION Ghana Project (2014); Multivariate multiple regression analysis

*p<1.0

**p<0.05 (estimations based on follow-up data only)

**Legend:** GAR = Greater Accra Region; WR = Western region; Risk area1 = Leadership processes and accountability; Risk area 2 = Competent and capable workforce; Risk area 3 = Safe environment for staff and patients; Risk area 4 = Clinical care of patients; Risk area 5 = Improvement of quality and safety

^+^Overall score = summated scores of all risk areas per clinic. Refer to Methods section for computation and interpretation of risk areas.

Post estimation predictive margins and contrast tests confirmed that on the whole, intervention facilities have higher predictive probabilities of attaining better assessment scores than control facilities (p = 0.0299). Health facilities that are privately owned or located in Western region seemed to have benefited more from the interventions than government-owned facilities or Greater Accra region facilities (see Table 3).

**Table 3:** Post estimation of predictive margins.

		Delta-method			
Variables	Margin	Std. Err.	P>z	[95% Conf.	Interval]
**Intervention**					
Control clinics = 0	42.1	3.5	0.000**	35.4	48.9
Treated clinics = 1	48.5	3.6	0.000**	41.4	55.4
**Region**					
WR = 0	46.1	3.9	0.000**	38.4	53.7
GAR = 1	44.5	3.2	0.000**	38.2	50.8
**Intervention/Region**					
0 0	47.0	5.4	0.000**	36.6	57.4
0 1	37.1	4.6	0.000**	28.3	46.2
1 0	45.1	5.5	0.000**	34.3	55.9
1 1	51.7	4.5	0.000**	42.9	60.5
**Ownership**					
Public = 0	43.5	4.2	0.000**	35.3	51.7
Private = 1	47.0	3.0	0.000**	41.1	53.0
**Intervention/Ownership**					
0 0	45.7	5.3	0.000**	35.4	56.1
0 1	38.5	4.6	0.000**	29.6	47.4
1 0	41.2	6.2	0.000**	29.0	53.5
1 1	55.6	4.0	0.000**	47.8	63.3
**Post estimation Contrast**		**Delta-method**			
	**Contrast**	**Std. Err.**	**p-value**	**[95%Conf.**	**Interval]**
Intervention vs control	10.3	4.7	0.0299*	1.0	19.6
GAR vs WR	-1.2	5.1	0.8081	-11.3	8.8
Private vs Public	4.0	5.3	0.4562	-6.5	14.5

**Source:** WOTRO-COHEiSION Ghana Project (2014); Contrast significant (*p<0.05); predictive margins significant (**p<0.001) (estimations based on follow-up data only)

**Legend:** GAR (Greater Accra Region); WR (Western region)

### Associations between community groups and post-intervention risk assessment scores

**Fig 5:**
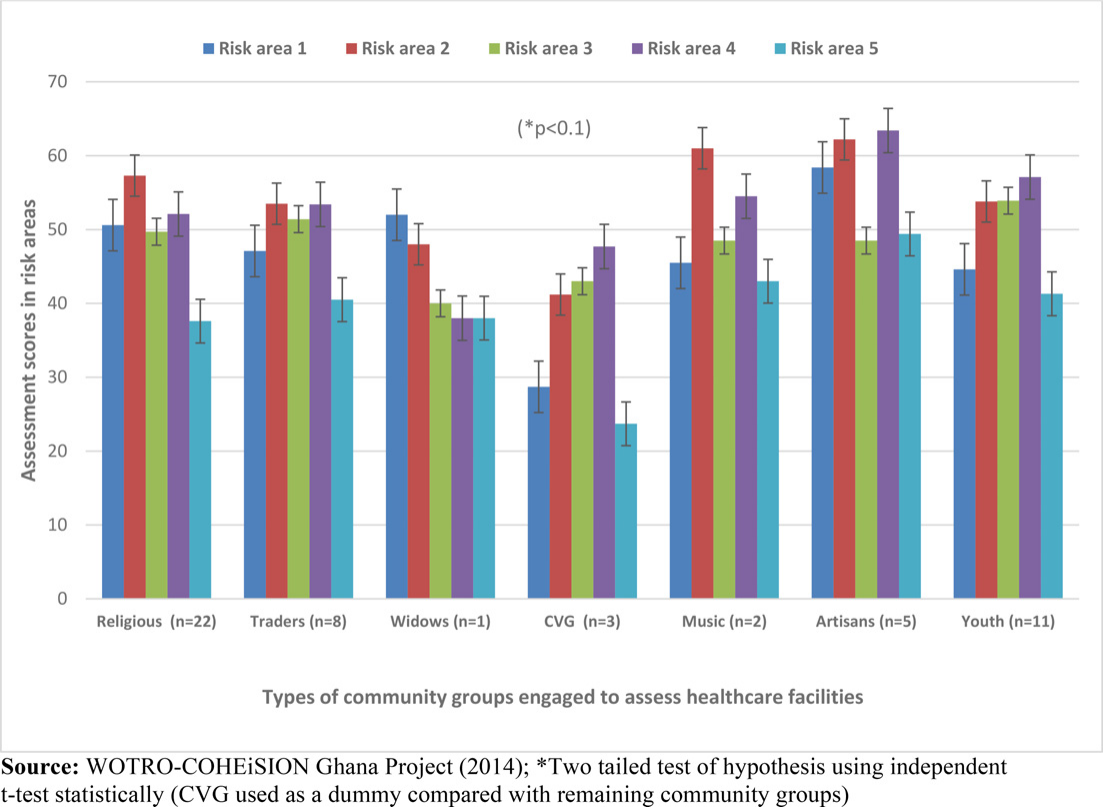
Types of community groups and post-intervention risk assessment scores. **Legend:** CVG (Community Volunteer Group); Risk area1 = Leadership processes and accountability; Risk area 2 = Competent and capable workforce; Risk area 3 = Safe environment for staff and patients; Risk area 4 = Clinical care of patients; Risk area 5 = Improvement of quality and safety

The results showed that the types of community groups involved in SCE have some significant associations with post-intervention risk assessment scores of healthcare facilities. As shown in Fig 5, community volunteer groups (CVGs) seemed have the least influence on assessment scores. Healthcare facilities assessed by CVGs attained mean% scores of 28.7 and 41.2 in risk areas 1 and 2 respectively compared to facilities assessed by other groups (p<0.1).

Spearman correlation test established a negative association between CVGs and assessment scores in risk area 1 (i.e. leadership and accountability) (Coef. = -0.2494, p<0.1) (see Table 4). Healthcare facilities that recorded the highest mean% scores in risk area 1 were assessed by artisans (mean = 58.4); traders (mean = 53.5); widows (mean = 52.0) and religious/faith-based groups (mean = 50.6) (see Table 4).

**Table 4:** Associations between types of groups and risk assessment areas.

Community characteristics		Risk areas					
		Risk area1	Risk area2	Risk area3	Risk area4	Risk area5	Overall^+^
**Group type**	**N**	**Coef.**	**Coef.**	**Coef.**	**Coef.**	**Coef.**	**Coef.**
Religious	22	0.1257	0.0942	-0.1651	-0.1961	-0.0887	-0.1144
Traders	8	-0.0256	-0.1290	0.0502	-0.0322	0.0357	0.0142
Widows	1	-0.0096	-0.1412	-0.0943	-0.1882	0.0094	-0.0468
Community volunteers	3	-0.2494*	-0.1442	-0.1499	-0.1385	-0.2156	-0.2204
Music	2	-0.0618	0.0941	-0.0135	0.0336	0.0402	0.0334
Artisans	5	0.2062	0.1886	0.2152	0.2936**	0.1487	0.2440*
Youth	11	-0.1035	-0.0506	0.1236	0.1803	0.0694	0.0755
**Gender distribution**							
All males	2	-0.0137	-0.0134	0.0404	-0.0336	0.0670	0.0267
All females	5	0.1703	0.0658	0.1449	0.0833	0.0787	0.1569
Male dominant	13	-0.3632**	-0.2956**	-0.3767**	-0.2954**	-0.3989**	-0.4212**
Female dominant	31	0.1804	0.1712	0.1741	0.1685	0.2312*	0.2068
Equal males and females	1	0.1540	0.1977	0.1979	0.1976	0.1690	0.2151
**Age distribution**							
Youthful (18–30 years)	18	-0.1944	-0.0082	-0.0027	0.1032	-0.0813	-0.0594
Elderly (31+ years)	34	0.1944	0.0082	0.0027	-0.1032	0.0813	0.0594
**Education**							
Mainly illiterates	11	0.1877	0.1108	0.1965	0.1076	0.1831	0.2170
Mainly literates	12	-0.2760**	-0.1595	-0.1782	-0.0767	-0.2356*	-0.2134
Literates and illiterates	29	0.0798	0.0442	-0.0104	-0.0234	0.0493	0.0026
**Location**							
Rural	28	-0.1644	-0.0363	0.0623	0.0052	0.0103	0.0155
Urban	24	0.1644	0.0363	-0.0623	-0.0052	-0.0103	-0.0155
**Leadership/Organization**							
Structured	39	0.3265**	0.3523**	0.2212	0.2029	0.1578	0.2462*
*Ad hoc*	13	-0.3265**	-0.3523**	-0.2212	-0.2029	-0.1578	-0.2462*
**Meeting dynamics**							
Group size (mean = 29)	52	0.0242	0.0818	-0.1191	-0.1097	0.0021	0.0084
Attendance rate (mean = 60%)	52	0.0055	-0.1423	-0.0050	-0.0290	-0.1187	-0.0311
^a^Meeting duration (mean = 41)	52	-0.1517	-0.1788	-0.1526	-0.0985	-0.0724	-0.1548
^b^Time per participant (mean = 1.3)	52	-0.1650	-0.2568	-0.0772	0.0000	-0.1616	-0.1732

**Source:** WOTRO-COHEiSION Ghana Project (2014)

**Note:** Spearman rank correlation test *p<1.0

**p<0.05. Estimations based on follow-up data only and pairwise correlation coefficients not adjusted for Bonferroni or Sidak.

**Legend:** Risk area1 = Leadership processes and accountability; Risk area 2 = Competent and capable workforce; Risk area 3 = Safe environment for staff and patients; Risk area 4 = Clinical care of patients; Risk area 5 = Improvement of quality and safety

^+^Overall score = summated scores of all risk areas per clinic. Refer to Methods section for computation and interpretation of risk areas.

^a^Average duration of group meeting reported in minutes

^b^Average time for group member contribution reported in minutes

In terms of gender distribution of community groups, male dominated groups did not appear to favor improvement in healthcare quality after the interventions. As shown in Table 4, male only or male dominated groups had negative associations with all the risk assessment areas (p<0.05) relative to groups that are either female dominated or balanced male-female distribution.

It was also found that community groups with predominantly elderly members (30+ years) are more likely to influence positive change in leadership and accountability practices of health facilities than a relatively youthful group. Counter intuitively, community groups with mainly literate members associated negatively with all risk areas including risk areas 1 (Coef. = -0.2760, p<0.05) and 2 (Coef. = -2356, p<0.1).

In terms of geographic location, urban community groups were positively associated with better assessment scores in risk areas 1 and 2 but rural groups were not. Community groups that had structured leadership were positively associated with higher assessment scores in all the five risk areas including risk areas 1 (Coef. = 0.3265, p<0.05) and 2 (Coef. = 0.3523, p<0.05). Groups with large membership appeared to have negative association with scores in risk areas 3 and 4 but positively associated with risk areas 1, 2 and 5; longer meeting durations and contribution time per group member had negative associations with all risk areas (see Table 4).

## Discussion

Systematic engagement of communities in healthcare quality assessment is a potentially useful quality improvement strategy required to promote health provider accountability to clients and regain the dwindling client confidence in Ghana’s healthcare system [26,29,30]. The findings of this study corroborate conclusions in previous studies that when health providers realize their activities are closely monitored by the communities they serve, they turn to show greater accountability and responsiveness to clients’ needs [8–10,30–33]. Moreover, findings of this RCT suggest that harnessing available community resources through systematic engagement could complement central governments’ efforts towards quality improvement especially in primary healthcare facilities which constitute over 70% of the nearly 4,000 NHIS-accredited service providers in Ghana [25].

Even though the interventions did not seem to have significant effect on mainstream medical technical quality markers, the effect on administrative and non-technical components of healthcare was established. Perhaps limited client knowledge on medical technical processes of healthcare account for this outcome, albeit health literacy levels of the community groups were not explored via-a-vis their assessment scores in this study.

As demonstrated in this study, SCE is potentially cost effective and sustainable for health systems in Africa with limited resources because it is championed by community members and resources are mobilized from within the community. The SCE concept requires minimal financial commitment and can easily be sustained by communities with technical support from local health authorities.

Per the interventions design, approximately US$ 380.0 can be used to implement SCE cycle in a year. This modest financial commitment is potentially scalable considering the relatively higher monitoring and evaluation (M&E) costs undertaken by the GHS and NHIA in Ghana. In 2012 alone, the NHIA operating expenditure for M&E activities (including parliamentary M&E and district health projects support) was approximately GHC 11.10 million (over US$ 3 million equivalence) [25].

Besides the positive outcomes of this RCT, it is important to acknowledge that some results of the study are not necessarily attributed to the SCE interventions. First, the apparent large increases in health service utilization and improvements in some human and material resources in intervention clinics might be attributed to institutional and national level developments between the baseline and follow-up periods. This is because the SCE interventions had limited capacity to influence staff recruitments and resource allocation/distribution to these clinics. For instance, approximately 12% of the 32 intervention facilities were upgraded to higher levels before the follow-up survey and this might have correspondingly influenced the increased service utilization and human resource capacities of these facilities independent of the interventions (see Fig 3).

Even though the interventions did not explore religion and gender dynamics into detail, it appears from the findings that religious/faith-based groups; groups with gender balanced composition and groups with organized leadership are likely to be more suitable for effective implementation of SCE interventions. Although reasons for this outcome were not explored into detail, perhaps recommendations from these cadre of community groups were more respected by healthcare providers relative to other forms of community groups.

In addition, societal respect and trust for religious bodies in many local Ghanaian communities might explain this outcome. In a study involving 19 sub-Saharan African (SSA) countries including Ghana, Tortora [34] found that community members trusted faith-based organizations more than their governments. In light of this trust and confidence, these religious groups are increasingly being engaged in health programmes to guarantee successful implementation [35,36].

In terms of the gender dynamics of the community groups, it was found that engaging only males or male dominated groups did not have a positive association with healthcare quality assessment scores after the interventions, but groups which composed of males and females or were female dominated had a positive association with most quality care proxies. This observation could be explained by the fact that in Ghana (like many SSA countries) women, particularly in rural deprived areas, turn to have greater health needs than men [37,38]; moreover, women are more likely to utilize healthcare services and probably be conversant with quality care gaps than males [37].

Further exploration of these posits by future studies could help arrive at more concrete conclusions on gender dynamics in community engagement in healthcare quality assessment.

The negative association between community volunteer groups (CVGs) and healthcare quality assessment scores in all the five risk areas (see Table 4) appear to be counter intuitive given the valuable contributions of CVGs to health programmes at the primary healthcare level in many SSA countries [4,8–10] including Ghana [11,13,14,15]. This observation probably reveals a diminishing volunteering role of CVGs in health programmes at the community level.

Perhaps activities of CVGs are increasingly being undermined by the fast changing cosmopolitan societies in Africa and are no longer effective options for promoting community engagement in health. Since the current study did not explore these outcomes in detail, future studies could explore the role of CVGs in primary healthcare in contemporary health systems in Africa. This will help determine whether or not CVGs are indeed becoming defunct in primary healthcare interventions.

Furthermore, the evaluation found that community groups without formal education did not seem to constitute a barrier to assessing healthcare quality though lack of basic health knowledge might have limited their ability to adequately identify gaps in medical technical quality care components. Community groups that composed of mainly literate members associated negatively with high quality assessment scores in intervention facilities. Maybe group members with formal education had less contact and experiences with the primary healthcare facilities which are often perceived by the elite as “inferior” service providers [20].

In view of this, it is possible quality improvement recommendations from these so called educated groups were irrelevant to the lower level health facilities which they seldom patronize.

It is evident from the evaluation outcome that for community groups to be effective in SCE they must, *inter alia*, be better organized and perhaps heterogeneous in composition. The relevance of religious groups in ensuring effective implementation of community engagement activities is also implicit though not adequately explored in this study.

## Limitations

This RCT is not a double blind randomization and the researchers’ knowledge of which health facilities received intervention had the potential to introduce bias in analysis of results at the facility level [22]. Cognizant of this potential bias, facility codes were used during the analysis to anonymize the facilities. Furthermore, key informants in health facilities were blinded to the intervention and control clinics to reduce potential for biased responses due to Hawthorne Effect [39].

Besides the impact of the SCE interventions, upgrading of some health facilities from clinic/health centre to full-fledged hospital status before the follow-up survey might have influenced health infrastructure, human resources, service utilization and potentially scores of the quality healthcare proxies. In view of this, multivariate multiple regression analysis and post estimation predictive margins and contrast tests were used to correct the possible effect of these extraneous factors.

## Policy Recommendations and Highlights

Based on the evaluation outcome of this study, the following highlights are proposed for policy consideration:

1. **Policy dialogues and stakeholder consultation:** Health policy makers at the national, regional and district levels should initiate policy dialogues and comprehensive stakeholder consultation on how to leverage potentials of existing community groups in healthcare quality assessment using the SCE concept; during these dialogues, opportunities and challenges in using religious and gender based organizations for quality care assessment should be explored.
2. **Building on existing frameworks:** The SCE concept should be integrated into existing health sector policy frameworks on quality improvement by harmonizing current M&E budget lines and checklists with the SCE implementation steps.
3. **Expanding platforms for community engagement in health:** Peer reviews among health facilities and assessment of health sector performance (organized by GHS and MoH in Ghana) should effectively include SCE sessions to serve as platforms for disseminating community grievances on quality care in health facilities, beyond the conventional patient satisfaction survey reports.
4. **Proposed steps to piloting the SCE concept:** The following proposed steps should be considered by policy makers to determine the feasibility or otherwise of using existing community groups to assess healthcare quality in health facilities:
  - *Step 1*: *A policy discussion and pilot study should be initiated to determine the feasibility of the SCE on a small scale*. *Possible implementation challenges such as inadequate eligible community groups; health provider and/or community apathy and political interference should be addressed during this stage*.
  - *Step 2*: *A SCE experiment in nationally representative communities should be conducted to inform a progression onto step 3 or otherwise*.
  - *Step 3*: *A replication of the SCE experiment in many districts in the country to ascertain whether or not it can survive in other settings with different demographic and socio-economic conditions*.
  - *Step 4*: *Once a replicated experiment attains significant success rate*, *a nationwide scale-up will then be warranted*.

## Conclusion

Systematic engagement of existing community groups in healthcare quality assessment has the potential to enhance efforts towards better administrative and accountability processes by healthcare providers but might not be effective in assessing mainstream medical technical quality due to possible knowledge/information asymmetry between clients and healthcare providers.

Even though the SCE interventions might have the challenge of community groups’ heterogeneity and its varying effect on healthcare quality assessment outcomes, the SCE initiative is deemed relevant and potentially sustainable for the Ghanaian context and other resource poor settings in SSA.

While acknowledging some limitations of this RCT, the authors conclude (based on the evaluation outcome) that community engagement in healthcare quality assessment is less expensive to implement and community-focused; thus, it should be discussed for piloting and eventual integration into existing quality improvement strategies in Ghana where universal access to basic quality care remains a major challenge [40]. Likewise, other SSA countries equally confronted with quality care challenges could learn from this evidence from Ghana and possibly replicate the SCE initiative in their health systems.

## Supporting Information

S1 Fig Interventions Design. (DOCX)

S2 Fig Light Engagement (LE) intervention implementation steps. (DOCX)

S1 Table Correlation between community groups’ characteristics and clinic features (n = 52). (DOCX)

## Abbreviations

AU: African Union
COHSASA: Council for Health Services Accreditation of Southern Africa
DHMTs: District Health Management Teams
ERC: Ethical Review Committee
GAR: Greater Accra Region
GHS: Ghana Health Service
JCI: Joint Commission Internationa
LE: Light Engagement
NHIA: National Health Insurance Authority
NHIS: National Health Insurance Scheme
RCT: Randomized Controlled Trial
SCE: Systematic Community Engagement
SSA: Sub-Saharan Africa
WHO: World Health Organization
WR: Western Region
