# Supplementary material for: Effect of Community Engagement Interventions on Patient Safety and Risk Reduction Efforts in Primary Health Facilities: Evidence from Ghana
Source: PLoS One. 2015 Nov 30;10(11):e0142389. doi: 10.1371/journal.pone.0142389 (PMC4664410; doi:10.1371/journal.pone.0142389)
Supplement: S1 Fig — (DOCX) [file pone.0142389.s001.docx]

**S1 Fig: Systematic Community Engagement (SCE) interventions design**

**Source:** WOTRO-COHEiSION Ghana Project (2014); **Legend:** GAR (Greater Accra Region); WR (Western Region); SCE (Systematic Community Engagement); LE (Light Engagement); n (sample size).

**NOTE:** *MyCare* intervention is not the focus of this paper thus it is not elaborated in this paper.
