# Supplementary material for: Effect of Community Engagement Interventions on Patient Safety and Risk Reduction Efforts in Primary Health Facilities: Evidence from Ghana
Source: PLoS One. 2015 Nov 30;10(11):e0142389. doi: 10.1371/journal.pone.0142389 (PMC4664410; doi:10.1371/journal.pone.0142389)
Supplement: S2 Fig — (DOCX) [file pone.0142389.s002.docx]

**S2 Fig: Systematic Community Engagement (SCE) Interventions (SCEIs) implementation steps**

**Assessment areas**

=>Quality of services in NHIA offices

=>Quality of services in NHIS-accredited clinics

3 months

6 months

**Source:** WOTRO-COHEiSION Ghana Project baseline and follow-up field data (2014); **Legend:** C=Client; P=Provider; I=Insure; NHIS (National Health Insurance Scheme); NHIA (National Health Insurance Authority)
