# Supplementary material for: Effect of Community Engagement Interventions on Patient Safety and Risk Reduction Efforts in Primary Health Facilities: Evidence from Ghana
Source: PLoS One. 2015 Nov 30;10(11):e0142389. doi: 10.1371/journal.pone.0142389 (PMC4664410; doi:10.1371/journal.pone.0142389)
Supplement: S1 Table — (DOCX) [file pone.0142389.s003.docx]

**S1 Table: Correlation between community groups’ characteristics and clinic features (n=52)**

|  | **Clinic characteristics** | | | | | |
| --- | --- | --- | --- | --- | --- | --- |
|  | **Ownership** | **Location** | **Region** | **No. consulting rooms** | **No. Laboratories** | **Staff: patient ratio** |
| **Groups characteristics** | **Coef.** | **Coef.** | **Coef.** | **Coef.** | **Coef.** | **Coef.** |
| Group type | 0.1090 | -0.2389 | 0.3851* | -0.2184 | -0.1218 | -0.1414 |
| Gender distribution | -0.05424 | 0.0880 | -0.0456 | -0.1146 | 0.0432 | -0.0006 |
| Age distribution | 0.0988 | 0.1871 | -0.1617 | 0.1766 | 0.0075 | 0.1899 |
| Education | -0.1043 | 0.1767 | -0.0477 | 0.1619 | -0.3174* | 0.1833 |
| Leadership/Organization | 0.0642 | 0.1782 | 0.0444 | 0.1053 | 0.0808 | -0.0528 |
| Group size (mean=29) | 0.5660* | -0.3307* | 0.2318 | 0.1764 | 0.0241 | -0.2752* |
| Attendance rate (mean=60%) | -0.1029 | 0.2328 | -0.0108 | -0.1282 | 0.0062 | 0.0669 |
| Meeting duration (mean=41) | 0.3219* | -0.2706 | -0.0007 | 0.1911 | -0.0292 | 0.1451 |
| Time per participant (mean=1.3) | -0.2562 | 0.2181 | -0.3051* | -0.1414 | 0.0320 | 0.3369* |

**Source:** WOTRO-COHEiSION Ghana Project (2014)

*Pairwise correlation test statistically significant (p<0.05)
